# The role of global economic policy uncertainty in long-run volatilities and correlations of U.S. industry-level stock returns and crude oil

**DOI:** 10.1371/journal.pone.0192305

**Published:** 2018-02-08

**Authors:** Honghai Yu, Libing Fang, Boyang Sun

**Affiliations:** School of Management and Engineering, Nanjing University, Nanjing, Jiangsu, China; Universitat Jaume I, SPAIN

## Abstract

We investigate how Global Economic Policy Uncertainty (GEPU) drives the long-run components of volatilities and correlations in crude oil and U.S. industry-level stock markets. Using the modified generalized autoregressive conditional heteroskedasticity mixed data sampling (GARCH-MIDAS) and dynamic conditional correlation mixed data sampling (DCC-MIDAS) specifications, we find that GEPU is positively related to the long-run volatility of Financials and Consumer Discretionary industries; however, it is negatively related to Information Technology, Materials, Telecommunication Services and Energy. Unlike the mixed role of GEPU in the long-run volatilities, the long-run correlations are all positively related to GEPU across the industries. Additionally, the rankings of the correlations of Energy and Materials are time-invariant and classified as high, with the little exception of the latter. The Consumer Staples industry is time-invariant in the low-ranking group. Our results are helpful to policy makers and investors with long-term concerns.

## Introduction

In recent years, much attention has been paid to the inter-relation among crude oil, stock market return, and economic policy uncertainty (EPU). EPU refers to the contribution of government policy makers to the uncertainty regarding fiscal, regulatory, or monetary policy [1]. It should be stressed that some industries are more sensitive to decisions driven by political events than others [2]. That is to say, EPU should drive the long-run volatilities of specific industry-level stock markets in different ways.

Furthermore, it is well known that sudden changes in global economic activity are important shocks driving the oil market and U.S. stock market [3–5]. The GEPU Index, proposed by [6], is a good international EPU index which can reflect the economic policy uncertainties from a global view. Through a human audit study and the comparison between EPU and other measures of economic and policy uncertainty, [1] showed that EPU was a reliable and accurate measure of national policy uncertainty. As a GDP-weighted average of the national EPU indices of 16 countries, GEPU can reflect the global condition of the global economy, uncertainty and policy-related matters. It is an appropriate measure of global policy uncertainty that market participants are really concerned about. The literature has shown a rising interest in EPU and GEPU. For example, [7] used EPU to investigate how corporate capital investment at the firm and industry levels were affected by the uncertainty related to future policy and regulatory outcomes. [8] applied the same method to create an index similar to EPU, using the Access World News database. [9] discussed how the GEPU index had recently spiked in three waves, and highlighted how it was both a cause and effect of recessions. Thus, one can expect the GEPU to drive the long-run correlations of specific industry-level returns to crude oil, in a specific manner.

Employing the modified generalized autoregressive conditional hetero-skedasticity mixed data sampling (GARCH-MIDAS), and dynamic conditional correlation-mixed data sampling (DCC-MIDAS) specifications, this study investigates the role of GEPU in long-run volatilities and correlations of the U.S. oil market and industry-level stock returns. To address concerns about robustness, the prices of crude oil are sourced from both the West Texas Intermediate (WTI) spot prices and the one-month futures contracts.

First, our empirical evidence contributes to the literature in terms of how GEPU drives the long-run volatility across industries. The volatility of the stock market has been demonstrated to be closely correlated to GEPU [1, 8, 10]. According to [1], EPU denotes the contribution of government policy makers to the uncertainty regarding fiscal, regulatory, or monetary policy. The rationale is that the uncertainty pertaining to economic policy decisions discourages firms’ investing activity and puts upward pressure on financing costs, which may also affect the expected cashflows and/or discount rates [11]. Thus, economic policy-induced uncertainty would significantly influence the volatility of the stock market. [12] and [8] show that EPU exerts a negative effect on stock market returns in 7 areas. [13] conclude that higher EPU would make stock returns more volatile and more correlated. [11] employ the DCC model to investigate the relationship between the EPU index and stock market return, and find that the stock return declines in response to increased EPU. In addition, [2, 14, 15] document a significant relationship between the volatility of the stock market (industry) and political uncertainty. [16] study the dynamic Evolution of Cross-Correlations in the Chinese stock market. The short-run volatility of the aggregated stock market driven by EPU has been widely discussed in the literature. However, unlike previous studies, we employ the EGARCH-MIDAS framework modified from the GARCH-MIDAS model proposed by [17] to investigate the impact of GEPU on the long-run volatility of the U.S. industry-level stock return. Specifically, GEPU is positively related to the long-run volatility of the Consumer Discretionary and Financial, and negatively related to Energy, Information Technology, Materials and Telecommunication Services.

The second contribution of the paper is revealing how GEPU drives the long-run correlation between crude oil and the industry-level stock market. A vast amount of literature focuses on the inter-relation of EPU, the stock market, and crude oil. Two recent papers are closely related to our work. [4] explore the relationship between structural oil shocks, EPU, and real stock returns. [18] use a modified DCC-MIDAS specification to show a long-run correlation with the macroeconomy. Another strand of the literature does not examine the impact of EPU, but documents the effect of oil price shocks on the stock market. Petroleum is the major energy source in the U.S., accounting for about 40% of the nation’s energy consumption [19]. [20] finds that both oil prices and oil price volatility play important roles in aggregated market return. At the industry level, [21] document that the excess return of the oil-using industries is more likely to be affected by the changes in the volatility of oil return than the oil return itself. Of course, the relationship between industry-level stock return and oil price shocks differs substantially [19, 22–24]. Their results indicate that not only industries that rely heavily on oil, but the stock returns of some industries that use lower volumes of oil are also sensitive to oil shock. [25] and [24] provide evidence in support of time-varying correlations between stock market prices and oil prices for different countries. These studies discuss the effect of oil shocks on industrial stock returns.

Our study is related to, but different from, these studies in the following two ways. First, we test the impact of GEPU on the long-run volatility of the industry-level returns, rather than aggregated market return. Then, we investigate the impact of GEPU on the long-run correlation between crude oil and industry-level stock returns. Employing the modified DCC-MIDAS specification [26], we find that GEPU has a positive impact on the long-run correlation between oil and industry returns in all cases, which is consistent with the finding of [13]. Further, the relationship between oil spot contract and industry is more vulnerable to GEPU than futures price. The correlation rises sharply during a crisis, slowly declines in the subsequent recovery period, and stays at a low level in the boom period, which is consistent with the results of [18]. Dividing the ranking of correlations into 3 yearly groups, we find that some industries, such as Energy and Materials, continuously show the highest correlation with both oil markets during the whole sample, but the rankings of the other industries change across time. The impact of GEPU on the relationship between most industries and the oil market is moderate and mixed.

The findings are helpful to practitioners and researchers. First, the long-run equilibrium correlation and economic policy fluctuations can be crucial to help policy makers understand the transmission mechanisms of their decisions and adopt policies accordingly. Furthermore, the results can also greatly improve the efficiency of asset allocation for portfolio managers and investors. Our paper shows that GEPU is a determinant of the long-run volatility, while volatility is a key input to derivative pricing and portfolio allocation problems. We also provide much-needed guidance to long-term investors who want to allocate part of their wealth to some specific industries. The industries significantly related to GEPU are more exposed to such risk, increasing their volatility accordingly. Finally, uncertainty pertaining to economic policy decisions would also affect firms’ investing activity. High levels of policy uncertainty can also cause households and businesses to hold back significantly on spending, investment, and hiring. Thus, it is necessary for managers to understand the effect of GEPU.

## Data description

We use daily WTI spot prices and one-month futures prices for crude oil, obtained from the Energy Information Administration (EIA). The data of one-month futures contracts are obtained from the New York Mercantile Exchange (NYMEX). The sample period spans from January 2, 1997 to April 18, 2016. The reasons for using the one-month futures contracts data are as follows. First, temporary random noise affects oil spot prices much more than the futures prices [20]. Second, the equity returns of firms operating in oil exploration, refinery, and marketing are co-integrated with the one-month and four-month oil futures prices [27, 28]. Third, as concluded by [21, 29], the variability of the futures prices is a judgment of the efficiency of hedging activities of firms engaged in hedging. However, considering the fact that the majority of previous studies have preferred using the spot price [30–32], we use both the spot and the futures prices data, similar to [33]. We believe that spot prices reflect information available to the markets up to time *t*, and futures prices measure the sentiment of the market participants towards the short-term future.

To quantitatively capture the degree of policy uncertainty in the world, we use the global economic policy uncertainty (GEPU) index, as in [6]. It is a monthly index, based on newspaper coverage frequency. The GEPU Index is a GDP-weighted average of the national EPU indices of 16 countries. Each national EPU index reflects the relative frequency of the own-country’s newspaper articles that contain a trio of terms pertaining to the economy, uncertainty and policy-related matters. Thus, we can consider that the monthly EPU Index value is proportional to the average share of newspaper articles that discuss economic policy uncertainty in that month [6]. Our GEPU data are consistent with [6] and are available at a monthly frequency on www.policyuncertainty.com.

We follow the Global Industry Classification Standard (GICS) system to collect the industry data. GICS was developed in response to the demand of the global financial community for one complete, consistent set of global sector and industry definitions, thereby facilitating seamless company, sector, and industry comparisons across countries, regions, and the globe. We collected 10 S&P500 GICS Level 1 Sector indices, which refer to capitalization-weighted companies. The data are available in the Bloomberg database for the same period as the data on crude oil. These 10 GICS industries are Consumer Discretionary (COND), Consumer Staples (CONS), Energy (ENRS), Financials (FINL), Health Care (HLTH), Industrials (INDU), Information Technology (INFT), Materials (MATR), Telecommunication Services (TELS), and Utilities (UTIL). We use the S&P500 GICS Level 1 Sector indices for several reasons. First, much attention has been paid to the SIC Sector indices and CRISP Level 1 Sector indices, but few studies have taken the S&P500 GICS Level 1 Sector indices into consideration. Second, S&P500 is one of the most commonly followed equity indices. It is popularly considered as one of the best representations of the U.S. stock market and economy. Thus, the industry indices we use are representative of the U.S. stock market and industries. Third, the S&P500 GICS Level 1 Sector indices are popular among practitioners, and consequently draw much interest.

Table 1 shows the summary statistics for the return series and GEPU. The sample means of returns are positive for both markets, which demonstrates that the U.S. economy grew slowly in these years. The standard deviation of crude oil (for both futures and spot prices) is around 2.5 and provides evidence for stronger fluctuations in crude oil than the stock market.

**Table 1:** Descriptive statistics of the data.

	Mean	Med	Max	Min	SD	Skew.	Kurt.	Obs
*COND*	0.0345	0.0758	12.3131	-10.0992	1.4049	-0.0386	8.9643	4830
*CONS*	0.0251	0.0448	8.8353	-9.2961	0.9903	-0.1084	11.0116	4830
*ENRS*	0.0239	0.0487	16.9604	-16.8836	1.6775	-0.2707	12.0893	4830
*FINL*	0.0100	0.0346	17.2013	-18.6390	1.9699	-0.0783	17.1897	4830
*HLTH*	0.0313	0.0565	11.7131	-9.1733	1.2291	-0.1341	8.8821	4830
*INDU*	0.0238	0.0594	9.5164	-9.2150	1.3652	-0.2535	7.7288	4830
*INFT*	0.0250	0.0932	16.0769	-10.0077	1.8255	0.1807	7.8895	4830
*MATR*	0.0161	0.0509	12.4730	-12.9339	1.5635	-0.1935	8.7081	4830
*TELS*	0.0027	0.0156	12.9261	-10.3203	1.4525	0.0651	9.3208	4830
*UTIL*	0.0135	0.0690	12.6840	-8.9962	1.2090	-0.0377	12.2113	4830
*OilFutures*	0.0089	0.0385	16.4097	-16.5445	2.4387	-0.0581	7.0002	4830
*OilSpot*	0.0098	0.0482	16.4137	-17.0918	2.5175	-0.0946	7.5050	4830
*GEPU*	1.0910	0.9971	2.8542	0.5008	0.4409	1.3226	5.1175	251

Table notes: The reported statistics are the daily logarithmic returns based on 10 GICS industry indices, and WTI crude oil spot and futures prices. The reported statistics include mean, minimum (Min), maximum (Max), standard deviation (SD), Skewness (Skew.), Kurtosis (Kurt.) and the number of observations (Obs). Daily WTI crude oil spot and futures prices data are collected from the U.S. Energy Information Administration. The 10 GICS industries are Consumer Discretionary (COND), Consumer Staples(CONS), Energy (ENRS), Financials (FINL), Health Care (HLTH), Industrials (INDU), Information Technology (INFT), Materials (MATR), Telecommunication Services (TELS), and Utilities (UTIL). Oil Futures and Oil Spot denote the results of futures and spot returns of crude oil, respectively.

The values of skewness for all 12 return series are around 0. The values of kurtosis are greater than 3 for all return series; however, it corresponds with the high-peak and fat-tail phenomenon of financial data series.

## Methodology

The model in this paper contains two sections. The first concerns the heteroscedasticity process of the return series, and we use the EGARCH-MIDAS model to investigate the impact on conditional volatility. The second concerns the dynamic correlation between the two assets. The DCC-MIDAS model is employed to analyze the impact of GEPU on long-run volatility and correlations between oil and industries.

### EGARCH-MIDAS model with GEPU

The EGARCH-MIDAS specification is modified to examine the effects of GEPU on the long-run volatilities of U.S. industry-level returns.

First, we consider the following mean equation of the returns on day *i* in month *t*,

$$\begin{array}{c} r_{i,t}=\mu_{i}+\sqrt{m_{t}\cdot g_{i,t}}\varepsilon_{i,t},\forall i=1,...,N_{t} \end{array}$$ (1)

where *ε*_*i*,*t*_|*ϕ*_*i*−1,*t*_ ∼ *N*(0, 1) with *ϕ*_*i*−1,*t*_ is the information set up to day (*i* − 1) of period *t*. Moreover, *g*_*i*,*t*_ and *m*_*t*_ are the short-run and long-run components, respectively.

Given the heteroscedasticity and the asymmetric effect of innovations, we modify the GARCH-MIDAS model proposed by [17] to EGARCH-MIDAS. The short-run component of *g*_*i*,*t*_ is assumed to be a (daily) EGARCH(1,1) process,

$$\begin{array}{c} \log g_{i,t}=u+\alpha (|\varepsilon_{i-1,t}\left|-E\right|\varepsilon_{i-1,t}\left|\right)+\gamma \varepsilon_{i-1,t}+\beta \log g_{i,t-1} \end{array}$$ (2)

The long-run component *m*_*t*_ is modeled as a slowly varying function of the lagged GEPU with the MIDAS specification.

$$\begin{array}{c} \log m_{t}=m_{l}+\theta_{l}\sum_{k=1}^{K_{l}}\varphi_{k}\left(\omega_{l}\right)X_{l,t-k}^{gepu} \end{array}$$ (3)

where $X_{l,t-k}^{gepu}$ is the *level* of *GEPU*.

The weighting scheme in the above specification is the so-called beta weight defined as

$$\begin{array}{c} \varphi_{k}\left(\omega_{l}\right)=\frac{{\left(1-k/(K+1)\right)}^{\omega_{l}-1}}{\sum_{j=1}^{K}{\left(1-j/(K+1)\right)}^{\omega_{l}-1}} \end{array}$$ (4)

One can think of Eq (3) in the context of regression models with a latent regress, enabling us to conduct the estimation through the maximization of the likelihood function.

### DCC-MIDAS model with GEPU

The returns of the industry-level stock market and crude oil are denoted as **r**_*t*_ = (*r*_1,*t*_, *r*_2,*t*_)′ where *r*_1,*t*_ and *r*_2,*t*_ represent the returns of the industry and crude oil market, respectively. *ϕ*_*t*−1_ = *σ*(**r**_*t*−1_, **r**_*t*−2_, …) is the *σ* field generated by the information available up to *t* − 1. Let *E*[**r**_*t*_|*ϕ*_*t*−1_] = *μ*_*t*_ = (*μ*_1,*t*_, *μ*_2,*t*_)′, and we define the vector of residuals filtered by Eq (1) as **r**_*t*_ − *μ*_*t*_ = *ε*_*t*_ = (*ε*_1,*t*_, *ε*_2,*t*_)′. The residuals have zero conditional mean, and we denote their conditional covariance matrix by **H**_*t*_ = **Var**[*ε*_*t*_|*ϕ*_*t*−1_].

The DCC model proposed by [34] is

$$\begin{array}{ccc} & r_{t}|\phi_{t-1}∼N\left(\mu_{t},H_{t}\right) & \\ & H_{t}=D_{t}R_{t}D_{t} \end{array}$$ (5)

where **D**_*t*_ is a diagonal matrix with standard deviations of returns on the diagonal, and **R**_*t*_ is the conditional correlation matrix of the standardized return residuals of the univariate return.

The standardized residuals are defined as *η*_*t*_ = (*η*_1,*t*_, *η*_2,*t*_)′ and have been already obtained from the EGARCH-MIDAS model, where $\eta_{t}=D_{t}^{-1}\varepsilon_{t}$.

Following [34], we avoid modeling the correlation matrices **R**_*t*_ directly, and specify the so-called “quasi-correlations”

$$\begin{array}{c} Q_{t}=\left(1-a-b\right)\bar{R_{t}}+a\eta_{t-1}\eta_{t-1}^{{}^{\prime }}+bQ_{t-1} \end{array}$$ (6)

where **Q**_*t*_ is the conditional covariance in matrix form. Therefore, the conditional correlation is estimated as

$$\begin{array}{c} R_{t}=diag{\left(Q_{t}\right)}^{-1/2}Q_{t}diag{\left(Q_{t}\right)}^{-1/2} \end{array}$$ (7)

The DCC-MIDAS model of [26] is a natural extension and combination of the DCC model and the EGARCH-MIDAS model. The long-run correlation component does not vary at the daily frequency *t* but at the lower frequency *τ*, both of which are shown in the EGARCH-MIDAS equation. That is, the short-run quasi-correlations fluctuate around the time-varying long-run correlations:

$$\begin{array}{c} q_{ij,t}=\left(1-a-b\right){\bar{\rho }}_{ij,\tau }+a\eta_{i,t-1}\eta_{j,t-1}+bq_{ij,t-1} \end{array}$$ (8)

where ${\bar{\rho }}_{ij,\tau }$ is the long-run component of the correlation.

We extend the DCC-MIDAS model by directly incorporating GEPU into the long-run component. Further, we apply the Fisher-z transformation of the correlation coefficient [35].

$$\begin{array}{c} {\bar{\rho }}_{ij,\tau }=\frac{\exp(2z_{ij,\tau })-1}{\exp(2z_{ij,\tau })+1} \end{array}$$ (9)

$$\begin{array}{c} z_{ij,\tau }=m_{ij}+\theta_{ij}\sum_{k=1}^{K_{c}}\varphi_{k}\left(\omega_{ij}\right)X_{l,t-k}^{gepu} \end{array}$$ (10)

Finally, the conditional short-run correlation is

$$\begin{array}{c} \rho_{ij,t}=\frac{q_{ij,t}}{\sqrt{q_{ii,t}}\sqrt{q_{jj,t}}} \end{array}$$ (11)

We use MATLAB software for our estimations. On the basis of this, we add the low-frequency GEPU into GARCH-MIDAS model. Given the widely recognized leverage effect in stock market returns, we have modified the original model into EGARCH-MIDAS to see the impact of GEPU on these industry-level returns.

## Empirical results

We provide the estimation results in the first subsection for the EGARCH-MIDAS model that relate the long-run volatilities to the GEPU index. In the last two subsections, we discuss the DCC-MIDAS specifications that focus on the long-run correlations and the rankings of the correlation, respectively.

### GEPU and the long-run volatility of industry-level returns

As described above, we incorporate global economic policy uncertainty (GEPU) into the EGARCH-MIDAS model to investigate the impact of GEPU on the long-run volatilities of the industries and the crude oil market. Table 2 presents the estimated results of the model, where *μ*, *α*, and *β*, are sourced from Eq (2), and *θ*, *ω*, and *m*, from Eq (3).

**Table 2:** Results of the EGARCH-MIDAS-GEPU model.

	*μ*	*α*	*β*	*u*	*γ*	*θ*	*ω*	*m*
*COND*	0.0228	0.1355***	0.9824***	-0.0991***	-0.1030***	0.2846*	12.2921	-0.3601
	(0.0171)	(0.0158)	(0.0043)	(0.0171)	(0.0120)	(0.1601)	(16.4221)	(0.6785)
*CONS*	0.0143	0.1601***	0.9766***	-0.1196***	-0.1043***	0.1433	33.9750*	-0.7601
	(0.0119)	(0.0163)	(0.0055)	(0.0168)	(0.0133)	(0.2168)	(20.6551)	(0.5304)
*ENRS*	0.0193	0.1198***	0.9838***	-0.0934***	-0.0764***	-0.7868***	1.0010***	1.4654**
	(0.0165)	(0.0140)	(0.0033)	(0.0159)	(0.0097)	(0.2945)	(0.0777)	(0.5948)
*FINL*	0.0083	0.1443***	0.9894***	-0.1099***	-0.0953***	0.3042*	3.7606***	0.0910
	(0.0166)	(0.0244)	(0.0032)	(0.0192)	(0.0122)	(0.1676)	(1.0386)	(0.4208)
*HLTH*	0.0109	0.1444***	0.9799***	-0.1097***	-0.0988***	-0.0320	12.8875***	0.0512
	(0.0133)	(0.0175)	(0.0045)	(0.0147)	(0.0128)	(0.2438)	(4.8931)	(0.5107)
*INDU*	0.0107	0.1212***	0.9826***	-0.0861***	-0.1056***	0.2200	3.2168**	-0.4032
	(0.0177)	(0.0148)	(0.0039)	(0.0128)	(0.0112)	(0.2874)	(1.3057)	(0.4934)
*INFT*	0.0252	0.1327***	0.9887***	-0.1075***	-0.0842***	-0.1944*	11.7810**	1.2900**
	(0.0159)	(0.0185)	(0.0030)	(0.0150)	(0.0115)	(0.1021)	(5.8961)	(0.6244)
*MATR*	0.0159	0.1290***	0.9853***	-0.0962***	-0.0730***	-0.5179*	1.0010***	0.8378
	(0.0152)	(0.0194)	(0.0037)	(0.0215)	(0.0107)	(0.2846)	(0.0298)	(1.0227)
*TELS*	-0.0005	0.1381***	0.9881***	-0.1020***	-0.0532***	-0.7895*	1.0010***	0.8112*
	(0.0108)	(0.0247)	(0.0035)	(0.0194)	(0.0104)	(0.4548)	(0.0756)	(0.4564)
*UTIL*	0.0199***	0.1904***	0.9794***	-0.1052***	-0.0467***	0.2495	41.0282***	-2.2897***
	(0.0071)	(0.0253)	(0.0051)	(0.0179)	(0.0114)	(0.2338)	(13.6925)	(0.5147)

Table notes: This table reports the estimates of the EGARCH-MIDAS coefficients for the considered assets. The 10 GICS industries are Consumer Discretionary (COND), Consumer Staples (CONS), Energy (ENRS), Financials (FINL), Health Care (HLTH), Industrials (INDU), Information Technology (INFT), Materials (MATR), Telecommunication Services (TELS), and Utilities (UTIL). The sample covers the period from 1997 until 2016. Robust standard errors estimated by sandwich algorithm when estimating covariance matrix in the maximum likelihood. The symbols “***”, “**” and “*” indicate statistical significance at the levels of 1%, 5% and 10% respectively.

The coefficients *α*, and *β* are highly significant in most industries. Moreover, for most industries, the coefficient *α* is slightly larger than that for the crude oil market, but the *β* coefficients are smaller. This means industries are more vulnerable to the innovation shocks but less sensitive to earlier lags. The sums of *α* and *β* are close to 1, indicating that the quasi-correlations are mean reverting in the long-run correlation. This finding is consistent with our expectation, and agrees with [17]. They noted that the sums of *α* and *β* are 0.96721 and 0.96085, respectively, for the fixed-span RV and rolling window RV cases.

The coefficient *θ* begins significantly positive means that GEPU significantly increases the long-run volatility of these industries. Considering Financials (FINL) as an example, the development of FINL is closely related to the economy, while GEPU is a counter-cyclical indicator. When GEPU is high, people will cut back investment and increase savings leading to a decline in liquidity, and higher risk and volatility.

However, the impact of GEPU on some industries, including Information Technology (INFT), and Telecommunication Services (TELS), are significantly negative. Firms belonging to INFT are mostly high-tech enterprises, their values depending on their products and technologies. Meanwhile, TELS is a necessary expenditure, and the cost of communication does not change much during recessions. Therefore, INFT and TELS do not register a sharp decline, while the other industries show rapid decreases during recessions. The volatilities of these two industries are also negatively related to GEPU.

There is a particular industry sector that deserves more explanation. Contrary to one’s intuition, Industrials (INDU) is more sensitive than other industries to oil price innovations induced by political events. One may expect GEPU to create volatility in these industries. However, our results show no significant relation in this regard. Since these industries are closely related to oil price changes driven by GEPU, they incorporate economic policy information quickly and efficiently. Thus, the long-run volatility is not vulnerable to GEPU. Our findings are similar to those of [33]. They conclude that industries directly related to the energy sector are so sensitive to oil prices that they do not predict oil shocks. The long-run volatilities driven by GEPU show minor fluctuations for these industries.

**Fig 1:**
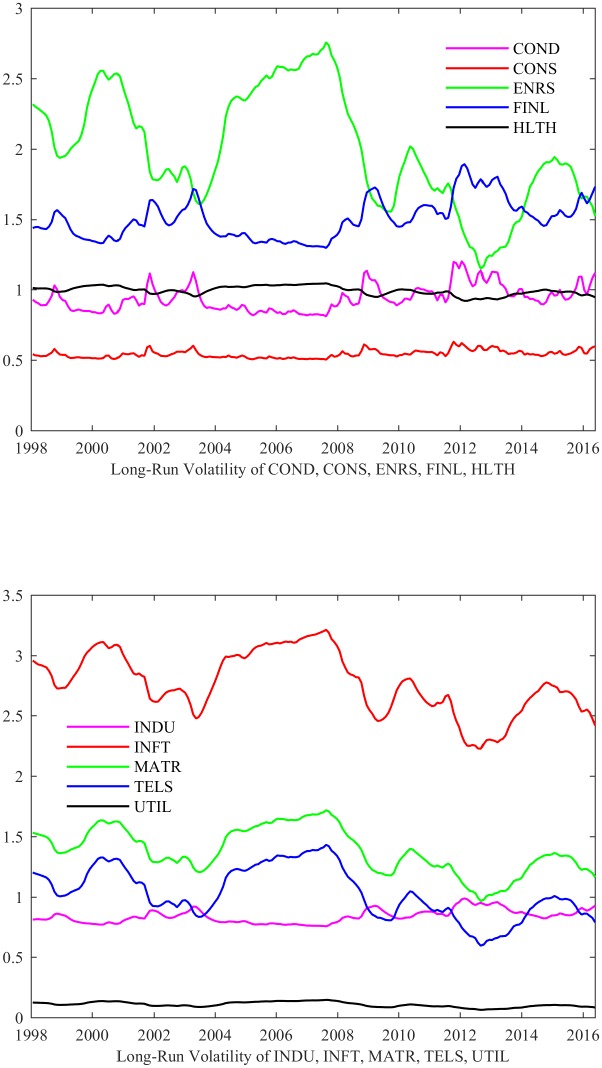
The long-run volatilities of the 10 industries. This figure presents the long-run volatilities of the 10 industries, estimated from the EGARCH-MIDAS model with GEPU. The 10 GICS Level 1 industries are Consumer Discretionary (COND), Consumer Staples (CONS), Energy (ENRS), Financials (FINL), Health Care (HLTH), Industrials (INDU), Information Technology (INFT), Materials (MATR), Telecommunication Services (TELS), and Utilities (UTIL). The data of the long-run volatilities of 10 industries are aggregated on a monthly frequency.

In sum, the estimated results of Table 2 imply that GEPU has a significant effect on the long-run volatility for more than half the industries. More specifically, Fig 1 displays the long-run volatilities of the 10 industries, which evolves smoothly.

### GEPU and the dynamic correlations between oil and industry-level returns

The second interesting finding of the paper concerns the effect of GEPU on the long-run correlation between oil and industry-level returns. The estimated results of the DCC-MIDAS-GEPU model are presented in Table 3. Similar to Table 2, the sums of the coefficients *α* and *β* are close to 1. Both parameters are significant in all industries and the oil market.

**Table 3:** Results of the DCC-MIDAS-GEPU model.

	Oil Futures					Oil Spot				
Industry	*α*	*β*	*m*	*θ*	*ω*	*α*	*β*	*m*	*θ*	*ω*
*COND*	0.0315***	0.9522***	-0.5151***	0.6038***	1.3819	0.0272***	0.9536***	-0.5253***	0.6175***	1.4981
	(0.0064)	(0.0118)	(0.0748)	(0.0588)	(4.1708)	(0.0065)	(0.0126)	(0.0717)	(0.0550)	(6.0663)
*CONS*	0.0209***	0.9684***	-0.4481***	0.5183***	1.3312	0.0202***	0.9648***	-0.5030***	0.5681***	1.6451**
	(0.0062)	(0.0124)	(0.0955)	(0.0744)	(3.5616)	(0.0066)	(0.0153)	(0.0823)	(0.0663)	(0.7783)
*ENRS*	0.0158***	0.9803***	0.1640***	0.4059***	49.3991***	0.0146***	0.9808***	0.1113*	0.4368***	49.7540***
	(0.0042)	(0.0060)	(0.0563)	(0.0338)	(2.2832)	(0.0050)	(0.0075)	(0.0632)	(0.0413)	(1.8754)
*FINL*	0.0208***	0.9710***	-0.6721***	0.7986***	1.0280***	0.0178*	0.9709***	-0.6867***	0.8014***	1.2532
	(0.0053)	(0.0086)	(0.0824)	(0.0624)	(0.2846)	(0.0092)	(0.0259)	(0.2039)	(0.1826)	(16.0052)
*HLTH*	0.0285***	0.9547***	-0.6201***	0.7062***	1.1730	0.0248***	0.9565***	-0.6423***	0.7240***	1.3506**
	(0.0054)	(0.0097)	(0.0610)	(0.0452)	(2.7393)	(0.0055)	(0.0103)	(0.0639)	(0.0513)	(0.5492)
*INDU*	0.0275***	0.9634***	-0.6038***	0.7628***	1.7480	0.0268***	0.9600***	-0.6187***	0.7698***	1.8402
	(0.0060)	(0.0088)	(0.0925)	(0.0715)	(1.5806)	(0.0063)	(0.0101)	(0.1039)	(0.0916)	(2.7842)
*INFT*	0.0245***	0.9647***	-0.4735***	0.6152***	1.5885	0.0224***	0.9649***	-0.4968***	0.6335***	1.5213
	(0.0054)	(0.0087)	(0.0657)	(0.0446)	(1.0357)	(0.0061)	(0.0105)	(0.1012)	(0.0908)	(1.9142)
*MATR*	0.0185***	0.9775***	-0.4805***	0.7438***	2.2778	0.0161*	0.9795***	-0.4706***	0.7294***	2.4670*
	(0.0071)	(0.0100)	(0.1091)	(0.0663)	(1.4079)	(0.0093)	(0.0144)	(0.1726)	(0.1202)	(1.3164)
*TELS*	0.0236***	0.9633***	-0.3719***	0.4496***	1.5426	0.0199***	0.9655***	-0.3753***	0.4498***	2.3664
	(0.0054)	(0.0103)	(0.0807)	(0.0758)	(3.6328)	(0.0056)	(0.0123)	(0.1393)	(0.1298)	(6.1070)
*UTIL*	0.0128***	0.9827***	-0.1417*	0.2821***	49.2450***	0.0115***	0.9839***	-0.1588**	0.3021***	49.2305***
	(0.0030)	(0.0049)	(0.0857)	(0.0588)	(4.1029)	(0.0031)	(0.0050)	(0.0674)	(0.0436)	(8.5963)

Table notes: This table reports the estimates of the DCC-MIDAS coefficients. The 10 GICS industries are Consumer Discretionary (COND), Consumer Staples (CONS), Energy (ENRS), Financials (FINL), Health Care (HLTH), Industrials (INDU), Information Technology (INFT), Materials (MATR), Telecommunication Services (TELS), and Utilities (UTIL). COND together with Oil Futures refers to the correlation between COND and the futures price of oil. COND together with Oil Spot denotes the correlation between COND and the spot price of oil, for example. The sample covers the period 1997 until 2016. Robust standard errors are used when estimating covariance matrix in the maximum likelihood. The number of MIDAS lags is 36 for the DCC process. The symbols “***”, “**” and “*” indicate statistical significance at the levels of 1%, 5% and 10% respectively.

The coefficient *θ* is estimated to be significantly positive for all 20 correlations, which means that GEPU drives the long-run correlations between oil and industry-level returns positively. Our findings are consistent with those of [13] that higher GEPU would lead to higher correlations in stock returns. The values of *θ* between the oil market and Utilities (UTIL) are the lowest among all 10 industries. This suggests that an increase in GEPU will raise the correlations between the oil market and utilities only slightly. The long-run correlations between this industry and the oil market show less fluctuation than the corresponding values for the other industries.

In addition, the values of *θ* for the correlations of industries to the oil futures market are of a similar magnitude, but slightly less than the spot market price in most industries. GEPU measures the degree of uncertainty with regard to economic policy. Spot price also contains the current uncertainty which oil market is exposed to. It could be expected that the correlations involving oil spot price are more vulnerable to GEPU, but the differences are small.

**Fig 2:**
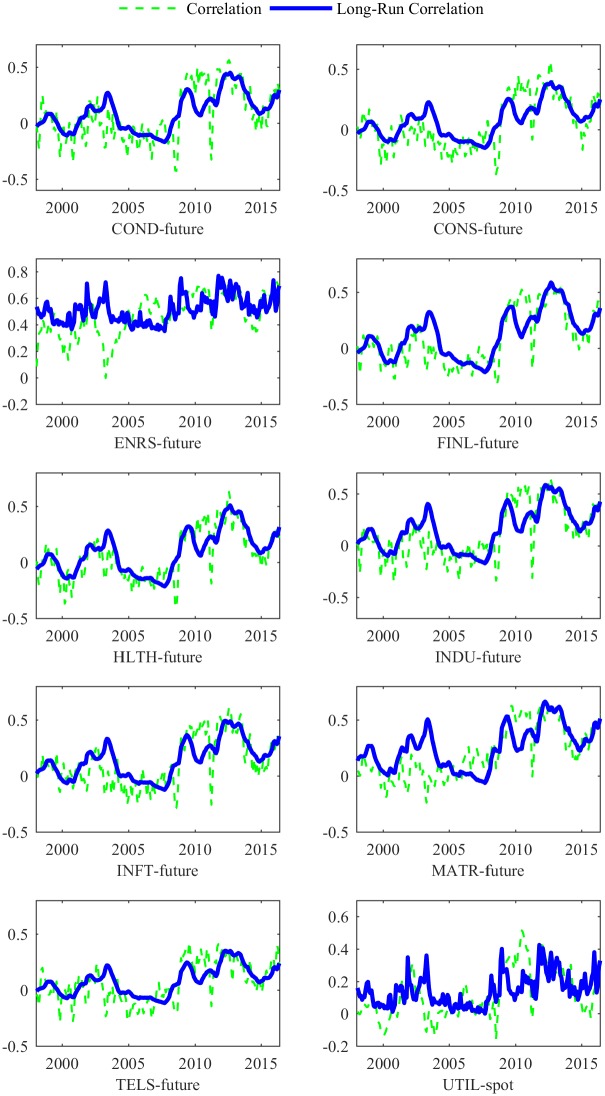
The long-run and total correlations of 10 industries and oil *future* price. This figure presents the long-run and total correlations of the 10 industries to the oil futures market, estimated from the DCC-MIDAS model with GEPU. The 10 GICS Level 1 industries are Consumer Discretionary (COND), Consumer Staples (CONS), Energy (ENRS), Financials (FINL), Health Care (HLTH), Industrials (INDU), Information Technology (INFT), Materials (MATR), Telecommunication Services (TELS), and Utilities (UTIL).

**Fig 3:**
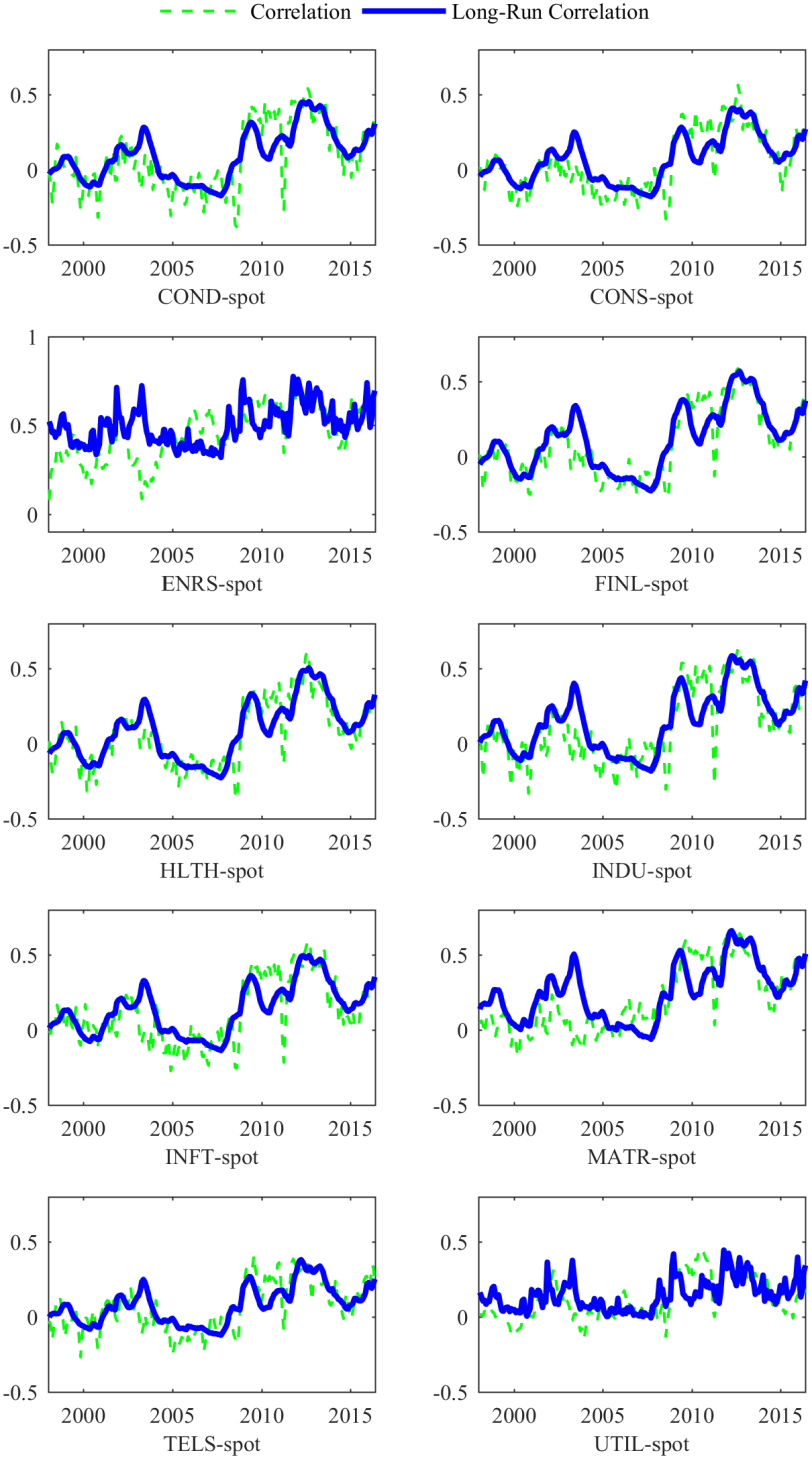
The long-run and total correlations of 10 industries and oil *spot* price. This figure presents the long-run and total correlations of the 10 industries to the oil futures market, estimated from the DCC-MIDAS model with GEPU. The 10 GICS Level 1 industries are Consumer Discretionary (COND), Consumer Staples (CONS), Energy (ENRS), Financials (FINL), Health Care (HLTH), Industrials (INDU), Information Technology (INFT), Materials (MATR), Telecommunication Services (TELS), and Utilities (UTIL) industry.

The estimated *θ* for the Energy (ENRS) industry is less than the average level (0.4059 for the correlation between ENRS and futures price, and 0.4368 for the correlation between ENRS and spot price). Since ENRS is closely related to crude oil, fluctuations in crude oil prices seriously affect the energy index. Therefore, GEPU has a smaller impact on the long-run correlation as an exogenous variable. Figs 2 and 3 show that the correlation between ENRS and the oil market ranges from 0.15 to 0.75 during the full sample. This indicates that oil price and the energy industry move in the same direction. This finding is consistent to our intuition that ENRS is so closely related to the oil market that GEPU drives little of the long-run correlation.

From 2004 to 2007, the total correlations between both oil markets and ENRS increase, while the long-run correlations decline at the same time. During this period, the ENRS index increases rapidly, as do the spot and futures prices of crude oil. The total correlations increase at the same time. However, U.S. economy grew rapidly with lower GEPU before the subprime crisis. This directly leads the long-run correlation between ENRS and oil market decline.

We provide the total and long-run correlations between the oil market and the 10 industries in Figs 2 and 3. Around the time of the recessions in 2000-2002 and 2007-2009, the long-run correlations between the oil market and the other 9 industries increase sharply from negative to positive. Then, during the recovery phases of the financial crisis, the oil–industry correlation slowly decreases back to the normal level. The Asian financial crisis in 1998 also causes the growth of relation. Since the Asian market has little impact on the US market, the effects last for a period less than the two recessions noted above.

Overall, an increase in GEPU would lead to growing concerns about future economic conditions, both for individuals and firms, and increase the long-run correlations. The long-run correlations are smoother than the total correlations, which fluctuate around the long-run trend. Furthermore, both correlations are relatively lower during the boom and recovery periods, but rise rapidly during the crisis phases (i.e., the Asian financial crisis in the late 1990s, the subprime crisis from 2007-2009, and China’s stock market crash in 2015).

### The ranking of dynamic correlations

In this section, we further investigate the time variation of the oil-industry correlations driven by GEPU. We also compare the differences across industries here. The time-varying correlation is not a necessary implication for the fluctuations in the relative ranking of relationships, but we find that the relative ranking changes across industries.

At first, we calculate the summary statistics of the long-run correlations between the oil market and industries for the full sample. Then, we provide the yearly rankings (the relationship between the industry and futures and spot prices, respectively) to investigate whether the rankings of the long-run correlations change constantly. The ranking is calculated as follows: In each year (period), we respectively rank the correlations to the oil futures and spot prices. Then, for each correlation, we calculate the number of times (relative to the whole sample) the correlation belongs to the “high group,” namely, where the ranking exceeds the 70th percentile; or the “low group,” that is, where the ranking is lower than the 30th percentile; or the “middle group,” containing the remaining values.

In Table 4, we demonstrate the results of the full sample and the yearly rankings of the long-run dynamic correlations. It clearly shows that not only correlations, but also relative rankings of some industries, fluctuate over time. However, Energy (ENRS) and Materials (MATR) show highly and persistent correlations with both oil spot and futures markets, since the correlations of these two industries both belong to the top percentile (100% of the full sample). The respective mean values of these two industries are 0.5147 and 0.2562 for the spot prices (0.4981 and 0.2526, respectively, for the futures price), which also agree with the observation made above. Furthermore, the correlation coefficient of ENRS is the highest among all industries. ENRS is too closely related to GEPU, which can incorporate economic policy information quickly and efficiently, and ENRS is highly related to the oil price; thus, the results pertaining to ENRS do not conflict with those in Table 2. Meanwhile, Consumer Staples (CONS) also shows a persistent, but the lowest, correlation with the oil market. This phenomenon is easy to understand. CONS provides the necessities of life. Irrespective of the kind of change in the oil market, people would not cut back their spending on this industry, making the relevant correlation the lowest of all.

**Table 4:** Descriptive statistics of the long-run dynamic correlations and their rankings.

	the correlation				Ranking of the correlation				Number of rankings		
Industry	Mean	Min	Max	Stdev	Mean	Min	Max	Stdev	High	Middle	Low
Panel A: The correlations of returns between industry and oil *spot*											
*COND*	0.0921	-0.1715	0.4487	0.1611	7.7059	7	8	0.4697	0	5	12
*CONS*	0.0739	-0.1532	0.3894	0.1396	8.3529	6	10	1.2217	0	5	12
*ENRS*	0.5147	0.3519	0.7702	0.0972	1.0000	1	1	0.0000	17	0	0
*FINL*	0.1258	-0.2148	0.5877	0.2048	5.9412	3	9	2.3841	4	8	5
*HLTH*	0.0888	-0.2158	0.5091	0.1860	8.0000	6	10	1.8708	0	9	8
*INDU*	0.1593	-0.1719	0.5816	0.1986	4.2353	3	7	1.2515	6	11	0
*INFT*	0.1435	-0.1253	0.4933	0.1625	4.4706	4	5	0.5145	0	17	0
*MATR*	0.2562	-0.0636	0.6634	0.1878	2.2353	2	3	0.4372	17	0	0
*TELS*	0.0814	-0.1172	0.3515	0.1222	7.8235	5	10	1.9441	0	7	10
*UTIL*	0.1444	-0.0002	0.4249	0.0947	5.2353	2	10	2.9692	7	6	4
Panel B: The correlations of returns between industry and oil *future*											
*COND*	0.0958	-0.1743	0.4539	0.1650	7.1176	6	8	0.4851	0	14	3
*CONS*	0.0698	-0.1803	0.4104	0.1540	8.7647	8	10	0.7524	0	0	17
*ENRS*	0.4981	0.3188	0.7752	0.1065	1.0000	1	1	0.0000	17	0	0
*FINL*	0.1159	-0.2281	0.5702	0.2081	6.2353	4	9	2.1369	0	11	6
*HLTH*	0.0852	-0.2281	0.5054	0.1914	8.1765	6	10	1.7761	0	7	10
*INDU*	0.1520	-0.1830	0.5855	0.2014	4.1176	3	7	1.4090	9	8	0
*INFT*	0.1385	-0.1378	0.4955	0.1670	4.7059	4	5	0.4697	0	17	0
*MATR*	0.2526	-0.0626	0.6609	0.1858	2.2353	2	3	0.4372	17	0	0
*TELS*	0.0796	-0.1228	0.3813	0.1257	7.7647	4	10	2.2508	0	6	11
*UTIL*	0.1474	-0.0073	0.4453	0.1010	4.8824	2	10	2.8258	8	5	4

Table notes: This table reports the summary statistics and yearly long-run correlations obtained from the DCC-MIDAS model. Panel A (B) reports the correlations of returns between industry and futures (spot) price of oil. The 4 columns to the left present the summary statistics for the estimated long-run correlations, and the 7 columns to the right show the yearly rankings of the correlations. The column with the number of rankings in each group presents the number of times the correlation ranking belongs to the “high,” “middle,” and “low” groups. The “high” (“low”) group contains the rankings of the correlations larger (lower) than the 70th (30th) percentile. The “middle” group contains the ones between the 30th and 70th percentile. The 10 GICS industries are Consumer Discretionary (COND), Consumer Staples (CONS), Energy (ENRS), Financials (FINL), Health Care (HLTH), Industrials (INDU), Information Technology (INFT), Materials (MATR), Telecommunication Services (TELS), and Utilities (UTIL).

In fact, except for the three industries above, the correlations and their rankings change significantly over time across industries, indicating that the impact of GEPU on the relationships between these industries and the oil market is moderate and mixed. For example, the standard deviations of the rankings for Financials (FINL) and Utilities (UTIL) are the largest among all industries. In Panel A, these two industries had, at some point in time, high (the top ranks being 3 and 2, respectively) and low (the lowest ranks being 9 and 10, respectively) correlations. Thus, the ranks of these industries are dispersed among all three groups. The standard deviations of the rankings of almost all these industries are larger than 1, which also proves that their rankings are changing constantly. Thus, although it is hard to distinguish between the rankings of these industries, most of them belong to the “middle” group. Besides, the correlation rankings between the industry and oil futures and spot prices share the same characteristics (see columns 6-9 in Table 4), which indicates that the correlation rankings are not affected by the futures or spot price of oil.

Our results show that some industries like Energy (ENRS), Materials (MATR), and Consumer Staples (CONS) have persistent correlation ranks during the whole sample. Specifically, ENRS and MATR are highly related to the oil market while CONS shows the lowest relationship. However, the rankings of most industries change across time. The impact of GEPU on the relationships between these industries and the oil market is moderate and mixed.

## Conclusion

We explore the impact of Global Economic Policy Uncertainty (GEPU) on the long-run volatility and correlation between the crude oil market and industry-level stock returns, based on the EGARCH-MIDAS and DCC-MIDAS models. A strand of the literature investigates the relationship between the oil and stock markets. However, the market-level analysis could mask differences among individual industries. Our findings are as follows.

First, we provide evidence that while GEPU is positively related to the long-run volatility of the Financials (FINL) and Consumer Discretionary (COND), it is negatively related to Information Technology (INFT), Materials (MATR), Telecommunication Services (TELS), and Energy (ENRS). Some industries, such as Consumer Staples (CONS), Industrials (INDU), Health Care (HLTH) and Utilities (UTIL), show insignificant links to GEPU. Secondly, GEPU has a positive impact on the long-run correlation between oil and stock returns in all cases. Considering oil spot prices, the relationship is more vulnerable to GEPU than the future price. Higher GEPU would lead to growing concerns about future economic conditions, both for individuals and firms, and an increase in the long-run correlation. The long-run or total correlations will stay low during a boom or recovery period, and rise rapidly during the recession phase. Finally, the rankings of the correlations clearly show that the Energy and Materials industries are time invariant in the high-ranking group, while the Consumer Staples industry is time-invariant in the low-ranking group. However, the rankings of most industries change over time, which means that the impact of GEPU on these correlations is mixed.

The findings of this study are helpful to both practitioners and researchers. The long-run equilibrium relationships of oil prices, stock markets, and economic policy fluctuations, can assist policymakers’ understanding of the transmission mechanisms of their decisions, and adopt policies accordingly. Our findings also offer indications of considerable improvements in asset allocation efficiency, for portfolio managers and investors. The industries significantly related to GEPU have more exposure to such risk, and thus, are more volatile. As economic activity is impacted by global economic policy, uncertainty pertaining to global economic policy decisions would also impact firms’ investing activity. Thus, it is necessary for managers to understand the effect of GEPU.

## Supporting information

S1 File The minimal underlying data. This is the package of supporting files. In this package, the readers can find the primary dataset in the XLSX/XLS files. The description of the data is also provided.
(ZIP)
