# Supplementary material for: The role of global economic policy uncertainty in long-run volatilities and correlations of U.S. industry-level stock returns and crude oil
Source: PLoS One. 2018 Feb 8;13(2):e0192305. doi: 10.1371/journal.pone.0192305 (PMC5805266; doi:10.1371/journal.pone.0192305)
Supplement: S1 File — This is the package of supporting files. In this package, the readers can find the primary dataset in the XLSX/XLS files. The description of the data is also provided. (ZIP) [file pone.0192305.s001.zip › Minimal Underlying Data/Explanation for raw data.docx]

All excel files in this folder contains our original index data and final result.

The file ‘Global Policy Uncertainty Data’ contains the ***monthly data of GEPU***, which are available on website [www.policyuncertainty.com](http://www.policyuncertainty.com).

The file ‘SP500 Industry Index’ contains ***10 S&P500 GICS Level 1 Sector indices*** which are constructed by capitalization-weighted from related companies. The data is available at Bloomberg database with the same period of crude oil. These 10 GICS industries are Consumer Discretionary (COND), Consumer Staples (CONS), Energy (ENRS), Financials (FINL), Health Care (HLTH), Industrials (INDU), Information Technology (INFT), Materials (MATR), Telecommunication Services (TELS), and Utilities (UTIL) industry.

The file ‘Crude Oil Price’ contains daily ***WTI Spot Prices and one-month futures prices*** ***for crude oil***, obtained from the EIA. The data of one-month future contact is obtained from New York Mercantile Exchange (NYMEX).

The file ‘Daily Return Statistics’ contains daily logarithmic returns based on ***10 GICS industry index and WTI crude oil spot and future price*** respectively.

The file ‘The long-run volatilities of 10 industries’ contains our results of long-run volatilities of which we have already drawn ***Figure 1*** in the paper.

The files ‘The long-run correlations between 10 industries and oil futures price’, ‘The total correlations between 10 industries and oil futures price’, ‘The long-run correlations between 10 industries and oil spot price’ and ‘The total correlations between 10 industries and oil spot price’ contain our results of long-run and total correlations of which we have already drawn ***Figure 2 and Figure 3*** in the paper.

All results are calculated by ***matlab*** program.
